# The Gender-Dependent Association between Obesity and Age-Related Cataracts in Middle-Aged Korean Adults

**DOI:** 10.1371/journal.pone.0124262

**Published:** 2015-05-14

**Authors:** Deok-Soon Lee, Kyungdo Han, Hyun-Ah Kim, Sae-Young Lee, Young-Hoon Park, Hyeon Woo Yim, Kang-Sook Lee, Won-Chul Lee, Yong Gyu Park, Kyung-Sun Na, Yong-Moon Park

**Affiliations:** 1 Graduate School of Public Health, The Catholic University of Korea, Seoul, Korea; 2 Yeoeuido St. Mary's Hospital, The Catholic University of Korea, Seoul, Korea; 3 Department of Preventive Medicine, College of Medicine, The Catholic University of Korea, Seoul, Korea; 4 Department of Biostatistics, College of Medicine, The Catholic University of Korea, Seoul, Korea; 5 Department of Ophthalmology, The Catholic University of Korea, Yeouido St. Mary’s Hospital, Seoul, Korea; 6 Division of AIDS, Center for Immunology and Pathology, National Institute of Health, Korea Centers for Disease Control and Prevention, Seoul, Korea; 7 Department of Epidemiology and Biostatistics, Arnold School of Public Health, University of South Carolina, Columbia, South Carolina, United States of America; Tufts University, UNITED STATES

## Abstract

The aim of this study was to investigate the association of central and abdominal obesity with the prevalence of cataracts in a middle-aged Korean population. This retrospective cross-sectional study was based on the data collected from the Korea National Health and Nutrition Examination Survey 2008–2009, in which 4,914 subjects were examined. Ophthalmological examinations were performed to determine the presence of a nuclear, cortical, or posterior subcapsular cataract. Both general obesity (a body mass index ≥25 kg/m^2^) and abdominal obesity (a waist circumference ≥90 cm for men and ≥80 cm for women) were significantly associated with the occurrence of cataracts among middle-aged women [adjusted odds ratio (aOR), 1.32; 95% confidence interval (CI), 1.03–1.69; and aOR, 1.40; 95% CI, 1.06–1.85, respectively], while abdominal obesity was significantly inversely associated with the occurrence of cataracts among middle-aged men (aOR, 0.76; 95% CI, 0.58–1.01; and aOR, 0.66; 95% CI, 0.49–0.89, respectively). We report a difference in the association between obesity and the prevalence of cataracts based on gender.

## Introduction

The development of age-related cataract is a major health concern worldwide. Cataracts are the leading cause of blindness, and account for approximately 51% of cases [1]. The prevalence of cataracts appears to be increasing with recent reports ranging from approximately 4.9% in Brazil to as high as 23% in Indonesia and Sweden [2–4]. It is expected that age-related cataracts will continue to be an important global health issue due to increasing life expectancy [5].

Previous studies have reported an increased risk of cataracts not only in individuals with general obesity[6,7] but also in those with abdominal obesity [8]. Certain studies have reported a stronger association of cataracts with abdominal obesity compared to general obesity, especially in women [9]. The association between obesity and the individual subtypes of cataracts has also been investigated. In some studies, a higher body mass index (BMI) was reported to be positively associated with cortical cataracts, but negatively associated with nuclear cataracts [10]. Recently, overweight group was shown to lower the risk of cataract significantly than the normal-weight group in Korean population [11].

Until now, reports on the association between cataracts and obesity have been inconsistent between studies. Moreover, most of the studies were conducted primarily using study groups of elderly people. Cataracts are often considered to be an unavoidable consequence of aging and aging is undoubtedly the strongest risk factor for the development of cataracts [12], thus the analysis of other factors that contribute to an increased prevalence of cataracts may be masked due to the age of the participants in these studies. In this study, we aimed to investigate the association of central and abdominal obesity with the development of cataracts in Korean adults with a separate analysis of middle-aged participants based on gender.

## Materials and Methods

### The study population

The fourth Korea National Health and Nutrition Examination Survey (KNHANES IV) comprises both independent and homogeneous annual rolling samples that were conducted between 2007 and 2009 in South Korea [13]. The sampling units were households selected through a stratified, multistage, probability sampling, based on geographic area, sex, and age group, of household registries. Information was collected from stratified multistage probability samples of Korean households representing the noninstitutionalized civilian population. The KNHANES was comprised of a health interview survey, a health examination survey, and a nutrition survey. The ophthalmologic survey, including ophthalmologic interviews and examinations, was introduced into the KNHANES IV in July 2008 for participants aged ≥3 years on behalf of the Korean Ophthalmological Society. Out of the 14,489 participants who were selected for the ophthalmologic survey in the second and third years of the KNAHENS IV, a total of 14,606 participants (99.0%) completed the ophthalmologic survey. Among those participants, 4,930 participants (2,142 males and 2,788 females) aged 40 to 64 years were included in this study. This study was approved by the Institutional Review Board of the Catholic University of Korea (CUMC11U138).

### Patient data collection

The health interview survey was designed to collect data pertaining to the general characteristics of the participants and included questions regarding socio-demographic variables, and health and behavior variables. Occupation was categorized according to the fields of agriculture, forestry, and fishery or other fields. Education was categorized into elementary school education of ≤6 years or higher education. Monthly household income was classified as either falling in the lowest quartile of income or in the 3 higher quartiles, according to the information provided by the participants. Smoking status was divided into smokers (including ex-smokers) and non-smokers, and alcohol consumption status was divided into drinkers (including ex-drinkers) and non-drinkers. Regular exercise was defined as walking for >30 minutes at 1 time, at least 5 times a week. Participants were also categorized into 2 groups according to whether they had been exposed to an average of >5 hours of sunlight per day, whether they had engaged in outdoor activities in the past 10 years, and whether they had a family history of eye disease, such as glaucoma, cataracts, strabismus, blepharoptosis, and retinal disorders.

Blood pressure was measured 3 times after 5 minutes of rest, and the average of the second and third measurements was considered as the final blood pressure for use in our analysis. Venous blood samples were taken from the participants after fasting for at least 8 hours. Fasting plasma glucose, total cholesterol, high-density lipoprotein-cholesterol (HDL-C), and triglycerides (TGs) were measured by enzymatic methods using a Hitachi Automatic Analyzer 7600 (Hitachi, Tokyo, Japan). Hypertension was defined as a systolic blood pressure ≥140 mmHg, a diastolic blood pressure ≥90 mmHg, or taking medication for the treatment of hypertension. Diabetes mellitus was defined as a fasting blood glucose ≥126 mg/dL, being diagnosed as such by health care professionals, or taking insulin or antidiabetic medications.

Height, weight, and waist circumference (WC) were measured by specially trained examiners using standard anthropometric equipment with the participants wearing only a lightweight gown. WC was measured at the midpoint between the lowest rib and the iliac crest using a measuring tape. In this study, general obesity was defined as a BMI ≥ 25 kg/m^2^, and abdominal obesity was defined as a WC ≥ 90 cm for men and ≥ 80 cm for women, according to the criteria for obesity established by the West-Pacific region of the WHO [12].

All ophthalmologic examinations were conducted by ophthalmologists. Details regarding ophthalmologic examinations are provided elsewhere [13]. The slit-lamp (Haag-Streit, Koeniz, Switzerland) examinations were performed to check for the presence of cataracts using a microscope set straight ahead, the light source at 30 to 45°, at 10× magnification, and the light beam set at the maximum height with a narrow width. The slit-lamp beam was also set wide open to observe the transparency, turbidity, pigmentation, vacuolation, and nucleus of the lens to determine the type and the severity of the cataract, as well as to look for the presence of the intraocular lens. Lens opacity was diagnosed by trained ophthalmologists by using the Lens Opacity Classification System (LOCS) II system. The Lens Opacities Classification System II (LOCS II) was used to classify opacities until into 7 cortical (C0, Ctr, CI, CII, CIII, CIV, CV), 5 nuclear (NO, NI, NII, NIII, NIV), and 5 PSC (P0, PI, PII, PIII, PIV) grades of increasing severity, according to photographic standards. Aphakia or pseudophakia were also documented even though excluded in the current study. The pupils were dilated with 1% tropicamide/2.5% phenylephrine hydrochloride eye drops, and the presence of lens opacity was examined by slit-lamp biomicroscopy and evaluated according to LOCS II standard photographs. The severity or grade of lens opacity was not recorded, and only the subtype of cataract present, including nuclear, cortical, posterior subcapsular, and anterior polar cataracts, was noted. Participants were defined as having a cataract if they had a nuclear, cortical, or posterior subcapsular cataract in at least 1 eye.

### Statistical analysis

All statistical analyses were performed using the SAS (version 9.2; SAS Institute, Inc., Cary, NC, USA) survey procedure using sampling weights defined by the KNHANES to provide nationally representative estimates that were adjusted for the survey year to minimize the variations between survey years. All data are presented as proportions (standard error) for categorical variables. The general and clinical characteristics of the subjects were compared with the presence of obesity and cataracts, and according to the age group. In addition, we conducted stratified analyses to assess the effect modification by gender in the association between obesity and cataract. The effect modification by gender was also tested by entering each interaction term (gender*BMI and gender*WC) in the full model. Based on the Directed Acyclic Graph (DAG), age, smoking, drinking alcohol, income, education, occupation, and sun exposure were treated as confounders. In contrast, the effect of obesity on cataract might be through metabolic abnormalities including diabetes, dyslipidemia, and hypertension in terms of mediation effect in which controlling for these mediators may result in over-adjustment. From a clinical perspective, however, we wanted to compare the model without metabolic abnormalities and the model with metabolic abnormalities (S1 Fig).

Logistic regression analysis was performed to estimate the association between general and abdominal obesity with different types of cataracts according to age group and sex. The odds ratios (ORs) and confidence intervals (CIs) were calculated using 3 different models. Model 1 includes an estimate after adjustment for age, and model 2, an adjustment for age, income, occupation, smoking status, alcohol consumption status, education, and sun exposure. Model 3 was tested after further adjustment for diabetes mellitus, high cholesterol, and hypertension. A *P-*value <0.05 was considered to be statistically significant.

## Results

### The general and clinical characteristics of the study participants

The mean age of men and women were 50.3 and 50.1 years old, respectively. The distribution of age in the study was 50.7% in 40–49 year-old group, 37.4% in 50–59, and 11.9% in 60–64 in men; and 49.7% in 40–49, 37.5% in 50–59, and 12.8% in 60–64 in women. There were significant effect modifications of gender on BMI and WC (P = 0.047 and P = 0.0003, respectively) in the full model, which supported the rationale for stratified analyses by gender (data not shown). There were significant differences in residence, education, occupation, sun exposure, alcohol, smoking, total cholesterol, HDL-C, TGs, WC, the presence of diabetes mellitus, and the presence of hypertension between men and women in the generally obese subjects and the generally non-obese subjects. In addition, there were significant differences between men and women in the presence of cataracts, residence, education, income, occupation, marital status, smoking, total cholesterol, HDL-C, TGs, BMI, the presence of cardiovascular disease (CVD), the presence of diabetes, and the presence of hypertension between the abdominally obese subjects and the abdominally non-obese subjects (Table 1).

**Table 1:** General and clinical characteristics of the study participants according to gender.

Male						
	< 25 kg/m^2^	≥ 25 kg/m^2^	*P*	< 90 cm	≥ 90 cm	*P*
	(n = 1,272)	(n = 862)		(n = 1,502)	(n = 632)	
Residence (rural)	21.7(2.6)	23.2(2.8)	0.4239	21.8(2.6)	23.6(3)	0.4255
Education (≤ 6 years)	17.1(1.2)	14.3(1.3)	0.1113	16.1(1.1)	15.5(1.6)	0.7162
Income (lowest quartile)	11.4(1.1)	9.5(1.2)	0.2303	10.3(0.9)	11.3(1.5)	0.5381
Occupation (agriculture/forestry/fishery)	7.6(1.3)	9.1(1.5)	0.1809	7.7(1.3)	9.4(1.6)	0.2027
Single (yes)	90.3(1)	90.8(1.2)	0.7636	90.4(0.9)	90.8(1.4)	0.8319
Sun exposure (more than 5 hours per day)	39(1.8)	38.1(2.1)	0.7472	38.1(1.7)	39.9(2.4)	0.5188
Outdoor activities in the past 10 years (yes)	98.9(0.4)	98.5(0.5)	0.496	99.1(0.3)	98(0.8)	0.0859
Family history of eye disease (yes)	18.7(1.2)	21.6(1.6)	0.1351	18.9(1.2)	22.2(1.9)	0.1326
Ever a smoker (yes)	81.6(1.2)	79.2(1.6)	0.2457	79.6(1.1)	83(1.6)	0.1067
Alcohol consumption (yes)	20(1.3)	23.2(1.5)	0.1229	19.3(1.1)	26(2)	0.0026
Regular exercise (yes)	30.6(1.5)	30.7(1.8)	0.9547	32.2(1.5)	26.6(2.1)	0.0323
Routine health checkup in the past 2 years (yes)	63.5(1.6)	62.9(1.9)	0.8159	63(1.5)	64(2.1)	0.6965
Cardiovascular disease history (presence)	2.6(0.5)	3(0.6)	0.5847	2.7(0.5)	2.9(0.7)	0.82
Diabetes (presence)	9(1)	17.8(1.5)	<.0001	9.6(0.9)	20.1(2)	<.0001
Hypertension (presence)	31.1(1.6)	49.8(1.8)	<.0001	32.7(1.5)	53.1(2.4)	<.0001
Total cholesterol (≥ 200 mg/dL or on medication)	36.7(1.5)	48.8(2)	<.0001	37.3(1.4)	51.8(2.5)	<.0001
Low high-density lipoprotein-cholesterol	18.5(1.3)	33.8(1.7)	<.0001	19.8(1.2)	36.8(2.1)	<.0001
Triglycerides (≥ 150 mg/dL or on medication)	35.8(1.4)	56.8(2)	<.0001	37.7(1.3)	60.4(2.5)	<.0001
Body mass index (≥ 25 kg/m^2^)	5.9(0.7)	62.9(1.9)	<.0001			
Waist circumference (≥ 90 cm for males, ≥ 80 cm for females)				21.3(1.1)	87.9(1.4)	<.0001
Pseudophakia	21.2(1.7)	19.6(1.9)	0.4163	18.7(1.5)	25.1(1.8)	0.0004
Cataract (presence)	26.3(1.8)	25(2)	0.5241	25.7(1.8)	26.1(2.4)	0.8574
Cortical type	7(1.2)	5.6(0.9)	0.1896	7.3(1.2)	4.2(0.9)	0.0219
Nuclear type	14.5(1.5)	14.4(1.9)	0.9288	13.4(1.5)	16.9(2.3)	0.0943
Female						
	< 25 kg/m^2^	≥ 25 kg/m^2^	*P*	< 80 cm	≥ 80 cm	*P*
	(n = 1,862)	(n = 918)		(n = 1,412)	(n = 1,368)	
Residence (rural)	19.8(2.4)	23.8(2.7)	0.0402	17.8(2.5)	24.7(2.7)	0.0029
Education (≤ 6 years)	23.3(1.3)	39.2(1.7)	<.0001	18.2(1.3)	39.4(1.5)	<.0001
Income (lowest quartile)	13(1)	17.3(1.4)	0.0046	13.1(1.1)	15.8(1.2)	0.0677
Occupation (agriculture/forestry/fishery)	5.2(1)	8(1.4)	0.0082	4(0.8)	8.3(1.4)	<.0001
Single (yes)	83.6(1.1)	80.7(1.7)	0.12	83.8(1.2)	81.6(1.3)	0.1779
Sun exposure (more than 5 hours per day)	16.5(1.1)	21.8(1.9)	0.0017	14.3(1.1)	22.6(1.7)	<.0001
Outdoor activities in the past 10 years (yes)	97.8(0.6)	98.4(0.6)	0.24	97.7(0.7)	98.3(0.6)	0.3699
Family history of eye disease (yes)	23(1.2)	23.7(1.7)	0.7083	24.5(1.4)	21.9(1.3)	0.1686
Ever a smoker (yes)	6.7(0.7)	5.9(0.9)	0.4976	6.4(0.8)	6.5(0.8)	0.8837
Alcohol consumption (yes)	1(0.3)	2.9(0.7)	0.0016	0.9(0.3)	2.4(0.5)	0.0034
Regular exercise (yes)	27.4(1.2)	30.7(1.8)	0.0985	28.3(1.3)	28.8(1.5)	0.8181
Routine health checkup in the past 2 years (yes)	63.9(1.3)	59.5(1.8)	0.0438	63.6(1.5)	61.6(1.5)	0.3591
Cardiovascular disease history (presence)	1.3(0.2)	2.4(0.5)	0.0207	0.9(0.2)	2.6(0.4)	<.0001
Diabetes (presence)	6.4(0.7)	11.9(1.3)	<.0001	3.5(0.5)	13.3(1.1)	<.0001
Hypertension (presence)	20.3(1.1)	40.2(1.9)	<.0001	16.5(1.1)	37.8(1.6)	<.0001
Total cholesterol (≥ 200 mg/dL or on medication)	40.7(1.3)	51.9(1.9)	<.0001	38.7(1.5)	50.4(1.5)	<.0001
Low high-density lipoprotein-cholesterol	35.7(1.3)	51.4(1.9)	<.0001	32.9(1.6)	49.1(1.5)	<.0001
Triglycerides (≥ 150 mg/dL or on medication)	21(1)	38.6(2)	<.0001	17.9(1.1)	36(1.6)	<.0001
Body mass index (≥ 25 kg/m^2^)	27.2(1.5)	91.2(1.2)	<.0001			<.0001
Waist circumference (≥ 90 cm for males, ≥ 80 cm for females)				5.3(0.7)	60.7(1.8)	
Pseudophakia	20.6(1.7)	20.4(2.2)	0.9361	15.9(1.5)	26.1(1.7)	<.0001
Cataract (presence)	22.5(1.6)	29.5(1.9)	0.0005	19.3(1.6)	30.8(1.9)	<.0001
Cortical type	5.5(0.9)	7.5(1.1)	0.1044	5.2(1)	7.3(0.9)	0.0735
Nuclear type	13.3(1.4)	18(1.9)	0.0062	11(1.4)	19.2(1.9)	<.0001

Data expressed as % (SE).

Low high-density lipoprotein-cholesterol (HDL-C) = HDL-C<40mg/dL (males) and HDL-C < 50mg/dL (female), or on medication.

### The prevalence of cataracts in the study population

**Fig 1:**
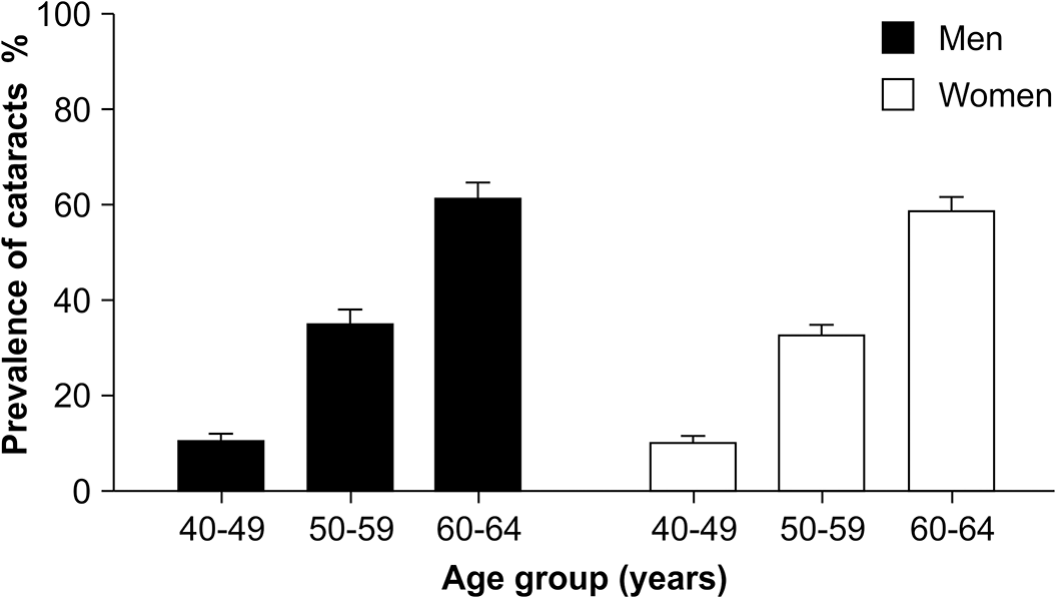
Age-specific prevalence of cataracts by gender. The error bars represent standard errors. The prevalence of cataracts increases with advancing age in both men and women.

**Fig 2:**
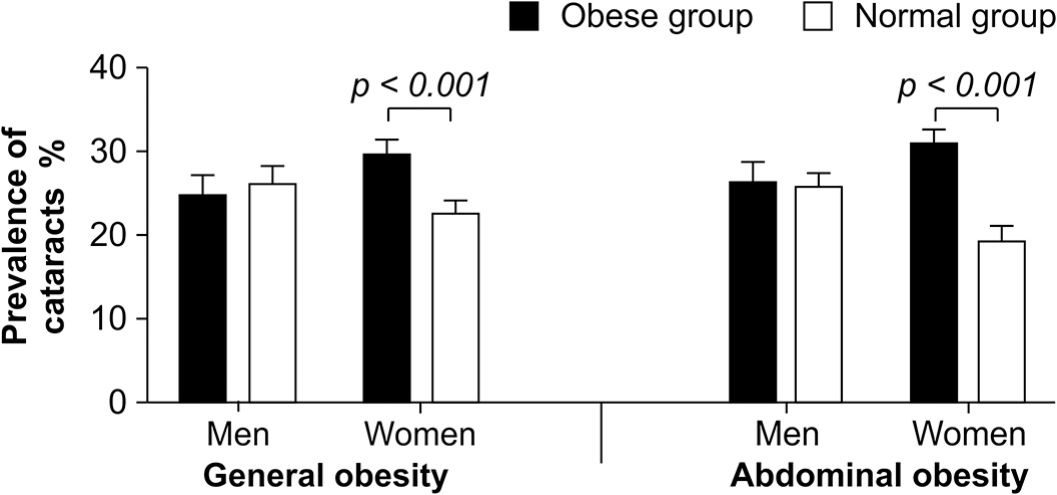
A comparison of the prevalence of cataracts according to age group and type of obesity. The prevalence of cataracts was significantly higher among middle-aged women in the generally obese group (29.5% vs. 22.6%) and the abdominally obese group (30.8% vs. 19.3%), compared with their non-obese counterparts (*P* < 0.001 for both types of obesity). General obesity = body mass index ≥ 25 kg/m^2^; Abdominal obesity = waist circumference ≥ 90 cm for men, ≥ 80 cm for women.

The prevalence of cataract increased with advancing age in both genders (Fig 1). The prevalence of cataracts was 25.0% among middle-aged participants (ages 40 to 64). Among men, 25.0% of the generally obese middle-aged group had cataracts. Of the abdominally obese adults, 26.0% of middle-aged men presented with cataracts. The prevalence of cataracts was 29.0% among the generally obese middle-aged women, and 35.0% among the abdominally obese middle-aged women. The prevalence of cataracts was significantly higher in both the generally obese group (29.0% vs. 23.0%) and the abdominally obese group (35.0% vs. 21.0%) compared with their non-obese counterparts (both *P* < 0.001) among middle-aged women (Fig 2).

### The comparison of middle-aged men and women according to the presence of cataracts

There were significant differences among the middle-aged male cataract group, and the normal group in residence, education, income, occupation, the amount of sun exposure, a family history of eye disease, total cholesterol, and the presence of CVD, diabetes, and hypertension. Among the middle-aged female participants, residence, education, income, occupation, marital status, the amount of sun exposure, CVD, diabetes, and hypertension, HDL-C, total cholesterol, TGs, and BMI, WC significantly differed between the cataract group and the normal group (Table 2).

**Table 2:** Univariate analsysis comparing between middle-aged men and women according to the presence of cataracts according to gender.

	Male			Female		
	Normal group	Cataract group	*P*	Normal group	Cataract group	*P*
Characteristic	(n = 1,490)	(n = 644)		(n = 1,995)	(n = 785)	
Residence (rural)	20.6(2.5)	27.1(3.8)	0.04	19.6(2.4)	25.6(3.6)	0.0531
Education (≤ 6 years)	12.3(1)	26.6(2.1)	<.0001	22(1.2)	47.2(2.3)	<.0001
Income (lowest quartile)	9(0.8)	15.2(1.7)	0.0001	21.1(1.9)	0(0)	<.0001
Occupation (agriculture/forestry/fishery)	6.7(1.1)	12.5(2.5)	0.0008	5.1(0.8)	9.1(2)	0.0013
Single (yes)	90.9(0.9)	89.6(1.6)	0.4487	83.9(1.1)	79.2(1.8)	0.0157
Sun exposure (more than 5 hours per day)	63.3(1.7)	55.8(2.3)	0.0064	84.4(1.1)	73.6(2.3)	<.0001
Outdoor activities in the past 10 years (yes)	99(0.3)	98(1)	0.2885	98.3(0.4)	97.1(1.3)	0.1891
Family history of eye disease (yes)	21.5(1.3)	15.1(1.7)	0.005	23.7(1.1)	21.8(1.7)	0.3448
Ever a smoker (yes)	81(1.1)	79.7(1.7)	0.5344	6.6(0.7)	5.9(0.9)	0.5194
Alcohol consumption (yes)	20.6(1.1)	23.1(2.1)	0.2922	*1*.*7(0*.*3)*	1.4(0.5)	0.6226
Regular exercise (yes)	31.2(1.4)	28.8(2)	0.3279	28.4(1.2)	29(2)	0.7582
Routine health checkup in the past 2 years (yes)	63.1(1.4)	63.8(2.2)	0.7985	62.3(1.3)	63.6(2)	0.5886
Cardiovascular disease history (presence)	1.9(0.4)	5(1.1)	0.0008	1.2(0.2)	3.2(0.6)	0.0001
Diabetes (presence)	10(0.9)	20.1(1.9)	<.0001	6.4(0.6)	13.4(1.4)	<.0001
Hypertension (presence)	36.7(1.5)	44.6(2.5)	0.0079	22.6(1.1)	38.6(2.6)	<.0001
Total cholesterol (≥ 200 mg/dL or on medication)	42.3(1.5)	39.6(2.1)	0.2986	40.4(1.3)	56(2.1)	<.0001
Low high-density lipoprotein-cholesterol	39(1.4)	40.2(2.3)	0.6675	54(1.3)	62.9(2)	0.0003
Triglycerides (≥ 150 mg/dL or on medication)	44.1(1.5)	45(2.2)	0.726	23.5(1.1)	35.6(2)	<.0001
Body mass index (≥ 25 kg/m^2^)	41.2(1.4)	39.5(2.1)	0.5216	29.6(1.2)	37.7(2)	0.0006
Waist circumference (≥ 90 cm for males, ≥ 80 cm for females)	29(1.4)	29.5(2.2)	0.8632	43.7(1.4)	59(2.5)	<.0001
Pseudophakia	.	79.7(2.0)	.	.	83.7(1.6)	.

Data are expressed as % (SE).Low high-density lipoprotein-cholesterol (HDL-C) = HDL-C < 40 mg/dL (males) and HDL-C < 50 mg/dL (females), or on medication.

### The gender association between obesity and cataracts in middle-aged participants

General obesity was associated with cataracts among middle-aged women (Table 3). When compared with non-obese, middle-aged women, generally obese middle-aged women were more likely to have cataracts as determined using models 1, 2, and 3 (OR, 1.33; 95% CI, 1.05–1.69; OR, 1.34; 95% CI, 1.06–1.70; and OR, 1.32; 95% CI 1.03–1.69, respectively) and were also more likely to have a nuclear cataract as determined using model 2 (OR, 1.34; 95% CI, 1.01–1.77).

**Table 3:** The association between obesity and cataract types according to gender.

	Male		Female	
	(n = 2,134)		(n = 2,780)	
Cataract type	BMI	WC	BMI	WC
Any				
Model 1^a^	0.84 (0.65–1.09)	0.77 (0.58–1.03)	1.33 (1.05–1.69)	1.34 (1.02–1.78)
Model 2^b^	0.78(0.59,1.02)	0.69(0.52,0.93)	1.34(1.06,1.7)	1.37(1.03,1.82)
Model 3^c^	0.76(0.58,1.01)	0.66(0.49,0.89)	1.32(1.03,1.69)	1.4(1.06,1.85)
Cortical				
Model 1^a^	0.88 (0.57–1.34)	0.49 (0.30–0.82)	1.19 (0.79–1.81)	1.10 (0.68–1.77)
Model 2^b^	0.87(0.55,1.37)	0.49(0.29,0.82)	1.15(0.76,1.75)	1.09(0.66,1.78)
Model 3^c^	0.82(0.52,1.31)	0.44(0.25,0.76)	1.27(0.82,1.97)	1.16(0.72,1.85)
Nuclear				
Model 1^a^	0.94 (0.67–1.33)	1.11 (0.78–1.59)	1.30 (0.97–1.74)	1.30 (0.90–1.86)
Model 2^b^	0.82(0.57,1.18)	0.95(0.66,1.36)	1.34(1.01,1.77)	1.35(0.93,1.96)
Model 3^c^	0.79(0.54,1.15)	0.91(0.63,1.31)	1.29(0.96,1.75)	1.36(0.94,1.98)

Data are presented as OR (95% CI).

ORs were tested using logistic regression analysis.

Abbreviations: BMI, body mass index; WC, waist circumference; OR, odds ratio; CI, confidence interval.

^a^ Model 1, Adjusted for age

^b^ Model 2, Adjusted for age, income, occupation, smoking, drinking, education, and sun exposure

^c^ Model 3, Adjusted for age, income, occupation, smoking, drinking, education, sun exposure, diabetes, and high cholesterol, hypertension

The presence of cataracts was also related to abdominal obesity among middle-aged participants, but with contrasting patterns between men and women. Similar to generally obese middle-aged women, abdominally obese middle-aged women were more likely to have cataracts as determined using models 1, 2, and 3 (OR, 1.34; 95% CI, 1.02–1.78; OR, 1.37; 95% CI, 1.03–1.82; and OR, 1.40; 95% CI, 1.06–1.85, respectively) compared to abdominally non-obese middle-aged women. In contrast, it was shown using models 2 and 3 that abdominally obese middle-aged men were less likely to have cataracts (OR, 0.69; 95% CI, 0.52–0.93; and OR, 0.66; 95% CI, 0.49–0.89, respectively), and using models 1, 2, and 3 that this group was also less likely to develop cortical cataracts (OR, 0.49; 95% CI, 0.30–0.82; OR, 0.49; 95% CI, 0.29–0.82; and OR, 0.44; 95% CI, 0.25–0.75, respectively) when compared to abdominally non-obese middle-aged men.

## Discussion

In this study, we found that both general and abdominal obesity were significantly associated with cataracts only in middle-aged Korean women. In contrast, there was a significant inverse association between abdominal obesity and both cataracts and cortical cataracts among middle-aged men. The prevalence of cataracts was significantly higher among middle-aged women with general or abdominal obesity compared to non-obese women.

The association between obesity and cataracts has been previously investigated in numerous studies [6,8–10,14–17]. The observed association between general and abdominal obesity and cataracts among middle-aged women in this study was consistent with several previous large population-based studies. According to a prospective cohort study among American men and women aged ≥45 years, subjects with a BMI ≥ 30 kg/m^2^ had a 36% higher risk of developing cataracts compared to those with a BMI < 23 kg/m^2^ [6]. Another study conducted among women aged 53 to 73 living in the United States showed that weight and abdominal adiposity contribute to the risk of developing posterior subcapsular cataracts [17]. Additionally, in 1 prospective follow-up study, it was shown that after a 9-year follow-up of subjects who had no cataracts at baseline, a high BMI or waist-to-hip ratio (WHR) was associated with the development of cataracts [8]. Moreover, a stronger association of age-related eye disease with central obesity than with BMI among women has also been reported [9]. Although the association between obesity and cataracts in women has been validated in many previous studies, the present study showed such an association only among middle-aged women, suggesting an age-dependent association between obesity and the development of cataracts. Likewise, according to a study conducted in Sweden,[18] abdominal obesity was associated with an increased risk of cataract extraction, especially among women <65 years of age. Population-based study using identical KNHNES 2009 showed that the overweight group had significantly lower risk of any type of cataract (odds ratio, 0.70; 95% confidence interval, 0.50 to 0.97) in men and (odds ratio, 0.70; 95% confidence interval, 0.51 to 0.97) in women in the multiple logistic regression analyses. We used different age group because we aimed to eliminate the effect of aging in cataract formation.

One of the most interesting findings from this study was that central obesity among middle-aged men was inversely associated with the development of age-related cataracts, in particular, cortical cataracts. Moreover, although not statistically significant, BMI was also negatively associated with the development of cataracts in this group, indicating an inverse association between obesity and cataracts in middle-aged men. In the same context, there are also other reports of an association between a lower BMI and the development of cataracts. According to a population-based study in China, a lower BMI was associated with the development of all forms of cataracts and specifically, cortical cataracts [19]. In addition, the association between a lower BMI and nuclear cataracts has also been reported [20]. However, contrary to the findings of this analysis, a study conducted in a black population aged 40 to 84 years living in Barbados found an association between abdominal obesity and a high prevalence of cataracts [21]. Likewise, in another study conducted among men in the United States, both BMI and abdominal obesity were reported to be risk factors in the development of cataracts [8]. Although the reasons for the discrepancies in such findings are not known, it can be assumed that the association between obesity and cataracts varies among populations [22] and between genders. The differences between men and women regarding age-related eye diseases such as age-related maculopathy, has been attributed to hormonal effects [9].

Being underweight is often related to low socio-economic status, smoking, a history of diarrhea and dehydration,[23] and poor nutrition,[24] which are all risk factors for the development of cataracts. In contrast to most of the other reports from studies conducted in Asia, an analysis conducted in India showed no association between a lower BMI and age-related cataracts,[25] suggesting inconsistencies in the association even among Asians. Thus, further research regarding the relationship between being underweight and developing cataracts is required. The relationship between obesity and the different subtypes of cataracts has been analyzed in many previous studies [10, 16,26,27]. Although most studies on the subject have reported that obesity is associated with cortical and posterior subcapsular cataracts, certain studies have reported different results,[10] again indicating a need for further research in this field.

The present study has some limitations that warrant mentioning. First, this is a cross-sectional analysis and therefore, causality cannot be derived from the observed associations. Second, the study population was limited to South Koreans; thus, the results cannot be generalized to other populations. Lastly, we did not explore the association regarding the varying degree and duration of obesity. Further study should be needed to use linear or categorical variables to test the association of cataract and degree and duration of obesity. However, major strength of this study is that it is the first nationally representative population-based study in Korea to observe the relationship between obesity and the development of cataracts that has been confirmed through ophthalmologic examinations as a part of the KNHANES.

In conclusion, both general and abdominal obesity were significantly associated with the prevalence of cataracts among middle-aged Korean women, while a significant inverse association was found between abdominal obesity and cataracts and cortical cataracts among middle-aged Korean men. Different approaches may be required in the management of obesity according to gender for in the prevention of the development of cataracts among the middle-aged population.

## Supporting Information

S1 Fig Directed Acyclic Graph (DAG). Directed Acyclic Graph (DAG) presenting the relation between obesity and cataracts. Age, smoking, drinking alcohol, income, education, occupation, and sun exposure were treated as confounders.
(TIF)
