# Supplementary figures and images for: The Gender-Dependent Association between Obesity and Age-Related Cataracts in Middle-Aged Korean Adults
Source: PLoS One. 2015 May 14;10(5):e0124262. doi: 10.1371/journal.pone.0124262 (PMC4431863; doi:10.1371/journal.pone.0124262)

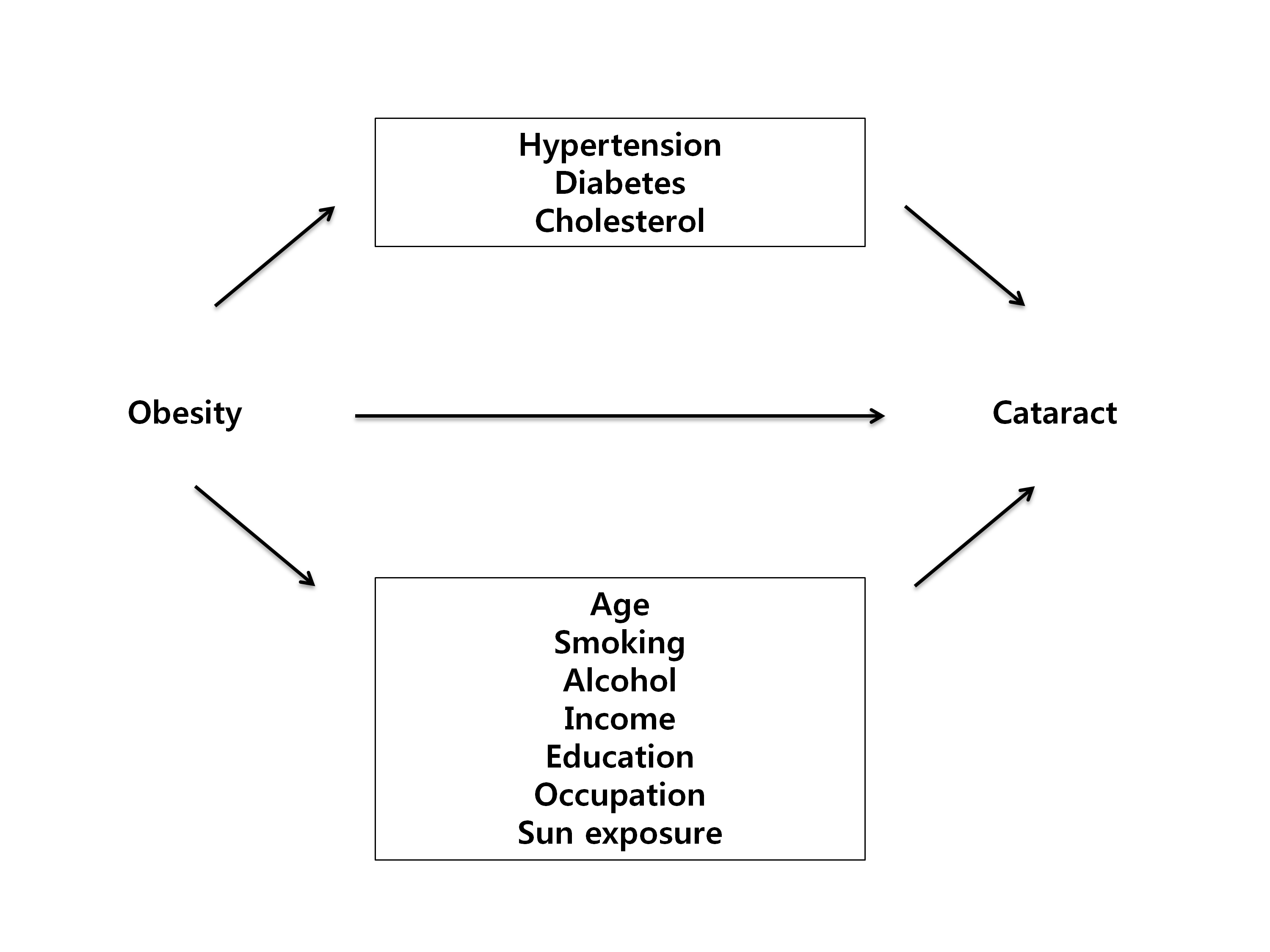

Supplement: S1 Fig — Directed Acyclic Graph (DAG) presenting the relation between obesity and cataracts. Age, smoking, drinking alcohol, income, education, occupation, and sun exposure were treated as confounders. (TIF) [file pone.0124262.s001.tif]
